# Acute Next-Day Effects of Alcohol Use on Daily Cognitive Functioning Among Young Adults: Protocol for a 21-Day Diary Study

**DOI:** 10.2196/77584

**Published:** 2025-09-17

**Authors:** Ashley N Linden-Carmichael, Jacqueline Mogle, Sara E Miller, Jennifer L Shipley, Stephen J Wilson

**Affiliations:** 1 Department of Counseling Psychology and Human Services University of Oregon Eugene, OR United States; 2 Prevention Science Institute University of Oregon Eugene, OR United States; 3 RTI Health Solutions Research Triangle Park, NC United States; 4 Edna Bennett Pierce Prevention Research Center The Pennsylvania State University University Park, PA United States; 5 Department of Psychology The Pennsylvania State University University Park, PA United States

**Keywords:** alcohol use, cognitive functioning, young adults, ecological momentary assessment, blackout drinking

## Abstract

**Background:**

Young adults exhibit the highest rates of binge drinking and heavy alcohol use of any age group. Blackout drinking, or alcohol-induced memory loss, is a negative consequence of heavy drinking and is common among young adults. Alcohol use has been found to affect postintoxication cognitive functioning, especially among those who have experienced blackout drinking. However, there have been a limited number of studies that have assessed alcohol use and cognitive functioning via ecological momentary assessment (EMA). This methodology allows researchers to have a better understanding of the association between alcohol use and next-day cognition as well as blackout drinking and potential moderators between these associations.

**Objective:**

The primary objective of the current study is to examine the association between alcohol use and next-day cognitive functioning among young adults. Multiple indices of alcohol use including blackout drinking and cognitive functioning will be examined. Additionally, moderators of these associations (at the day- and person-levels) will be examined. Potential moderators include other substance use, sleep, mood, hangover symptoms, and participant characteristics (eg, baseline alcohol use severity).

**Methods:**

Eligible participants had to be aged between 18-25 years, a current university or college student, residing in the Eastern time zone, endorsing heavy episodic drinking at least 2 times in a typical month, and reporting a blackout drinking episode at least once in the previous year. After completing a web-based screening survey, eligible participants were directed to a web-based baseline survey that asked about alcohol use, self-reported cognitive functioning, objective cognitive assessments, and demographic information. Participants were then sent 5 surveys a day for 21 consecutive days, which asked about substance use, sleep, mood, and self-reported and objective cognitive functioning. Multilevel models will be used to examine day-level associations between alcohol use and cognitive functioning.

**Results:**

A total of 304 participants were eligible, with 297 participants completing at least 1 EMA survey and cognitive assessment during the 21-day period. Data were collected between November 2023 and May 2024.

**Conclusions:**

The primary aim of this study is to examine the association between alcohol use (including blackout drinking) and next-day cognitive functioning, with a secondary objective of identifying potential day- and person-level moderators of these associations. Findings from the study may help inform momentary interventions and identify characteristics of young adults that may put them at higher risk for experiencing negative consequences.

**International Registered Report Identifier (IRRID):**

RR1-10.2196/77584

## Introduction

### Background

Rates of heavy alcohol use and alcohol use disorder peak in young adulthood around ages 18-25 years [1]. In 2023, an estimated 27% of young adults reported binge drinking (5+ drinks in a row) in the past 2 weeks, and 9% reported past 2-week extreme binge drinking or high-intensity drinking (10+ drinks in a row) [2]. An estimated 5.1 million young adults met criteria for a past-year alcohol use disorder in 2023 [1]. Even more common are acute negative outcomes of heavy drinking (eg, social-interpersonal consequences and adverse physical effects) [3]. A key negative consequence of heavy alcohol use is blackout drinking, or alcohol-induced memory loss during a drinking episode. Blackouts are common among young adults who consume alcohol, with approximately 50% reporting a lifetime history of a blackout [4]. Blackout drinking is particularly concerning given its associations with further harms from drinking, including physical injury, sexual assault, and overdose [5].

Controlled lab experiments have established the effects of alcohol use on postintoxication cognition in young adults [6,7]. Alcohol-related deficits have been identified in working memory [8], attention [9,10], psychomotor functioning [11,12], processing [13], and executive functioning [14]. Further, recent experimental evidence identified decreased volume in the posterior and central corpus callosum several days after one’s 21st birthday (ie, a single heavy drinking event), even after adjusting for lifetime alcohol and cannabis use, and these effects were more pronounced among individuals who experienced a blackout during the event [15]. While this evidence suggests a link between heavy drinking and structural brain damage postintoxication, it is unclear whether heavy alcohol use also impacts daily cognitive functioning, such as working memory and attention, among young adults. Given that changes to the corpus callosum impact communication between hemispheres, heavy drinking potentially reduces functional adaptation across regions, particularly under cognitive load [16]. Cognitive and brain reserve capacities describe the potential for individuals to compensate for changes in brain structure with learned strategies to avoid experiencing declines in cognitive functioning [17,18]. Hence, this initial evidence supports early theorizing on the role of substance use in altering cognitive and brain reserve capacity [19], suggesting that alcohol and other substance use may disrupt the development of this compensatory mechanism in young adults, ultimately leading to greater functional impairment.

As noted in Pearlson [20], a critical area of further exploration is whether young adults actually experience functional cognitive consequences or disruptions in general everyday cognitive functioning as a result of heavy drinking. Particularly for young adults whose brain can typically accommodate structural changes [21,22], structural brain alterations may be reversed in the long term due to the plasticity of their brain [22,23]. A more salient indicator may be younger individuals’ experiences of changes in everyday cognitive functioning [24]. Young adults who notice deficits in daily cognitive functioning may reduce engagement in cognitively stimulating activities (eg, social activities), establishing lifelong patterns of lower cognitive functioning.

Ecological momentary assessment (EMA), or collecting self-reported and behavioral data in real time and context via mobile devices, is ideal for studying functional changes in cognition. Disruptions in cognitive functioning can be small and are best captured via granular, sensitive measures in natural environments [25]. Functional changes can be observed in both objective measures (eg, executive functioning and memory) and subjective measures (eg, attentional lapses and difficulties concentrating). Beyond cognitive assessment, EMA has also been widely used for assessing alcohol use and its consequences among young adults [26,27]. Reporting on alcohol use in real time or the next morning can maximize recall of the amount of alcohol consumed [28]. Accurately identifying the number of drinks consumed in a drinking episode has important implications for identifying high-intensity drinking (8+ or 10+ drinks consumed in a day for female or male individuals, respectively) [29], which is associated with heightened risks for acute same- or next-day harms (eg, passing out or losing control) [30].

Analytically, EMA is ideal for examining the dynamic associations between alcohol use and cognition in daily life and for identifying contextual and person-level risk factors that may moderate such associations. EMA can be used to tease apart between- and within-person effects and thus isolate day-level effects of alcohol use on cognitive functioning on days with varying alcohol use intensity or experiences of blackout drinking. To date, very limited EMA or diary work has examined the role of alcohol use on cognitive functioning. In one study, higher intensity drinking was associated with difficulties in next-day self-reported attention and concentration [31], but performance-based measures of cognitive functioning were not assessed. Another EMA study examined the specific role of substance use on working memory across 7 days in a sample of young adults, finding that working memory was lower when using alcohol relative to not [32], but participants did not self-report experiences with their cognition. Among a sample of middle-aged and older adults who completed 8 days of surveys, participants self-reported their experiences with memory lapses and were more likely to have memory lapses if they drank more alcohol than the average amount they consumed [33].

As noted in prior EMA work, there is likely an acute impact of alcohol use on cognitive functioning that warrants further investigation, though several gaps in our understanding remain. First, with a more comprehensive assessment of cognitive functioning, we can determine whether the impacts of different levels of alcohol use are specific to a particular type of cognitive process (eg, working memory), global across cognitive processes (eg, fluid intelligence), or particular to an individual’s perceptions of their ability to recruit those processes to complete daily tasks (eg, self-reported cognitive functioning). Second, through multiple assessments throughout the day, we can explore the duration of the effect of alcohol use intensity on next-day cognitive functioning (eg, do effects occur only upon waking or persist throughout the day?). Third, given the unique risks of blacking out [5,34] and the more profound structural impairments linked to blackout episodes relative to heavy drinking without blacking out [15], research exploring blackout drinking and cognitive functioning is warranted.

### Aims and Objectives

#### Overview

Overall, even a single heavy drinking episode has been associated with short-term structural changes in the brain among a sample of young adults, and previous diary evidence suggests alcohol use may impact concentration and working memory capacity. As young adults are at higher risk of alcohol use and alcohol-related harms than any other age group, such immediate structural changes may signal a need for early prevention and intervention. Although the long-term impacts of alcohol use on the brain among young adults remain unknown, a key indicator of longer-term cognitive outcomes is observed functional impacts on young adult behavior in daily life. As such, changes in daily behaviors during this period could negatively impact cognitive health into midlife. In the current study, we collected intensive data across 21 days from a sample of young adults to explore the acute, next-day effects of alcohol use on cognitive functioning overall, across multiple timeframes, and by higher-risk drinking episodes (eg, high-intensity drinking and blackout drinking). We will also examine day-level and person-level moderators to identify moments and subgroups with greatest risk and greatest need for early targeted prevention and intervention efforts.

#### Primary Objective

The primary objective is to examine acute, next-day effects of alcohol use on cognitive functioning across a 21-day period for a sample of young adults. We will explore multiple indices of alcohol use (eg, whether they consumed alcohol and the number of drinks consumed) as well as higher-risk indices of alcohol use (eg, high-intensity drinking or blackout drinking) and their association with self-reported and objective measures of cognitive functioning (eg, daily memory lapses, episodic memory, and working memory).

#### Secondary Objectives

We will consider day- and person-level moderators of study associations between alcohol use and cognitive functioning to identify for whom and under what conditions the effects are most pronounced, which may ultimately inform targeted and real-time interventions for days and individuals at greatest risk. We will investigate other substance use, sleep, mood, hangover symptoms, and individual-level characteristics that have independent relationships with cognition or alcohol use [35-42] but have not yet been examined at the daily level among young adults who consume alcohol. Specifically, we will explore day-level moderators of hangover symptoms, mood, sleep, and prior-day cannabis and other substance use; and person-level moderators of sex, baseline alcohol use severity, and general cognitive functioning.

## Methods

**Participant Eligibility and Recruitment**

To be eligible in the current study, participants must have: (1) been age between 18 and 25 years, (2) been currently enrolled at a 4-year college or university, (3) resided in the Eastern time zone throughout the duration of the study, (4) endorsed engaging in heavy episodic drinking (4+ or 5+ drinks in one sitting for female or male individuals, respectively) at least twice during a typical month, and (5) reported blackout drinking at least once in the past year. To verify eligibility status, participants were required to provide a “.edu” email address (ie, an email address associated with a university) and had to be within the Eastern time zone to complete the baseline cognitive tasks (to ensure participants received EMA surveys at the correct times). Participants were recruited via (1) flyers posted on campus of a large, public university and in the surrounding community, (2) email invitations to prior research participants who expressed interest in future research studies, and (3) the StudyFinder database of research studies seeking volunteers. On the study flyer, potential participants were instructed to email the study team if they were interested in seeing if they were eligible to participate.

### Baseline Data Collection

Following informed consent, interested participants completed a brief web-based screener to determine eligibility. Eligible participants were automatically routed to complete a web-based baseline survey and cognitive assessments, which took approximately 30 minutes to complete. Participants completed a demographics survey; self-reported cognitive functioning items; performance-based cognitive assessments of episodic memory, executive functioning, and working memory; and self-reported typical alcohol use behavior (see Table 1 for the assessment battery). These measures represent key cognitive processes likely to impact daily functioning [43,44]. For episodic memory, participants completed a paired associates task: here, a list of 10-word pairs is presented, then participants are shown the first word of each pair and asked to recall the second. For executive functioning, participants completed a task-switching exercise categorizing objects by color (blue or green) or shape (square or rectangle) as quickly as possible based on a cue. For working memory, participants viewed 4 boxes that changed color in a random sequence and had to indicate when the same box changed colors twice in a row. Screening and baseline survey data were collected and managed with Research Electronic Data Capture (REDCap).

**Table 1:** Study assessment battery.

Construct and subdomains				Assessment
**Baseline assessment (approximately 30 min)**				
	**Cognition**			
		Episodic memory	Paired associates [45,46]	
		Executive functioning	Task switching [47,48]	
		Working memory	N-back [49,50]	
		Self-reported cognitive functioning	PROMIS (Patient-Reported Outcomes Measurement Information System) cognitive functioning [51]	
	**Alcohol use**			
		Quantity and frequency of alcohol use	Calendar grid of typical weekly drinking (Daily Drinking Questionnaire) [52]	
		Alcohol use severity	Alcohol Use Disorder Identification Test (AUDIT) [53]	
		Blackout drinking experiences	Alcohol-Induced Blackout Measure [54]	
	Demographic characteristics			Sex, age, race and ethnicity, and college education status
**Daily and momentary assessments (approximately 5 min/prompt)**				
	**Cognition (each prompt)**			
		Episodic memory	Modified backward number span [55,56]	
		Executive functioning	Modified Flanker inhibition task [57]	
		Working memory	Modified spatial n-back [58]	
		Self-reported cognitive functioning	Daily Cognitive Problems Checklist [43]	
	**Substance use (first daily report only)**			
		Substance use	Checklist: all substances used the previous day (eg, alcohol, cannabis, and nicotine). If alcohol was used, report the number of standard alcoholic drinks.	
		Blackout drinking	Whether endorsed, “I couldn’t remember what I did/Forgot what I did” in response to, “Did any of the following happen to you as a result of your alcohol/other substance use yesterday?” [59]	
	**Covariates or moderators**			
		Sleep quality (first daily report only)	Modified Pittsburgh Sleep Quality Inventory [60]	
		Hangover symptoms (each prompt)	Hangover Symptoms Scale [61,62]	
		Mood (each prompt)	Modified Positive and Negative Affect Schedule [63]	
		Motivation (each prompt)	Participants rate agreement with “I was motivated to perform well during this session.”	
		Current substance use (prompts 2-4 only)	Checklist of all substances used so far that day	

### Three-Week Ecological Momentary Assessment Data Collection

Follow-up surveys for the EMA portion of the study were collected online across 21 consecutive days (see Table 1 for a list of measures). Participants were sent email and text message prompts with the associated survey link daily at 11 AM, 1 PM, 3 PM, and 5 PM. The first prompt that participants completed each day included survey items about the prior day (“prompt 1”), including previous-day alcohol and other substance use, blackout drinking experiences, sleep, hangover symptoms, current mood, and cognitive functioning, plus objective (performance-based) assessments of cognition. At each subsequent assessment that day, participants reported on current substance use and mood and cognitive functioning was evaluated via both self-reported and objective cognitive measures. At the time of each prompt, participants were advised that they had 60 minutes to complete the survey questions and brief objective cognitive tasks. Average completion time per survey was approximately 3 (SD 6.12) minutes, and 90% of surveys were completed in less than 5 minutes. The EMA protocol was collected via Qualtrics. All objective cognitive assessments were collected and managed in the Gorilla Experiment Builder [64].

### Participant Incentives

Participants received up to $193 for full study participation. Specifically, participants received $10 for completing the baseline survey, $2 per prompt completed, and $5 per week bonus for completing at least 20 out of 28 surveys each week.

### Power Statement

We used simulations of planned multilevel models to account for dependencies in the data (SAS 9.4). For our primary objective (day- or moment-level associations between alcohol use and cognitive functioning outcomes), with n=300 and up to 84 surveys (21 days × 4 surveys per day), we have >80% power to detect relations between alcohol use and cognitive functioning accounting for 1% of variance. As some participants will not complete all surveys, we examined power for 80% and 50% compliance. These additional simulations resulted in power of >80% to detect the same relationship, indicating sufficient power in the presence of missing data. For secondary objectives, we are interested in differences due to day- and person-level moderators. To be conservative, we examined only scenarios with incomplete data (50% and 80%). Assuming these conditions, we can detect differences across moderators accounting for 1.7% and 1.6% of the variance, respectively.

### Ethical Considerations

The institutional review board at the Pennsylvania State University approved all study procedures (#STUDY00022639). Participants provided informed consent before enrolling in the study. Study data are deidentified.

## Results

Data were collected from November 2023 to May 2024. In total, 304 participants were eligible. Table 2 displays the demographic characteristics of the full sample. Of eligible participants, 97.7% (n=297) completed at least one EMA survey and cognitive assessment. Of participants who completed at least 1 EMA survey throughout the 21-day period (n=297), participants completed an average of 45 (SD 25.7) EMA surveys out of a possible 84 surveys. Average compliance for EMA surveys and cognitive assessments by prompt was as follows: 55.7% (SD 0.3%) for 11 AM, 57.1% (SD 0.3%) for 1 PM, 54.3% (SD 0.3%) for 3 PM, and 52.9% (SD 0.3%) for 5 PM. Of all possible days (6237 days), participants completed at least 1 daily survey on 73% (n=4553) of days. All primary data analyses are expected to be completed in 2026 and all primary manuscripts are expected to be submitted by 2027.

**Table 2:** Demographic characteristics of the participants (N=304).

Variable				Value
	**Biological sex (n=304), n (%)**			
		Male	64 (21)	
		Female	240 (79)	
	**Self-identified gender (n=304), n (%)**			
		Man	64 (21.1)	
		Woman	236 (77.6)	
		Gender nonconforming	1 (0.3)	
		Nonbinary	2 (0.7)	
		Transgender man	1 (0.3)	
	**Sexual orientation (n=304), n (%)**			
		Heterosexual or straight	254 (83.6)	
		Gay	5 (1.6)	
		Lesbian	2 (0.7)	
		Bisexual	30 (9.9)	
		Queer	6 (2)	
		Asexual	1 (0.3)	
		Pansexual	1 (0.3)	
		Questioning	5 (1.6)	
	Age (years), mean (SD)^a^			20.34 (1.25)
	**Age (n=304), n (%)**			
		Younger than 21 years	161 (53)	
		21 years or older	143 (47)	
	**Race (n=303), n (%)**			
		American Indian or Alaska Native	1 (0.3)	
		Asian	22 (7.3)	
		Black or African American	10 (3.3)	
		White	254 (83.8)	
		Not listed	5 (1.7)	
		Multiracial	11 (3.6)	
	**Ethnicity (n=303), n (%)**			
		Hispanic	23 (7.6)	
		Not Hispanic	280 (92.4)	
	**Race and ethnicity (n=303), n (%)**			
		Not Hispanic American Indian or Alaska Native	0 (0)	
		Hispanic American Indian or Alaska Native	1 (0.3)	
		Not Hispanic Asian	21 (6.9)	
		Hispanic Asian	1 (0.3)	
		Not Hispanic Black or African American	7 (2.3)	
		Hispanic Black or African American	3 (1)	
		Not Hispanic White	242 (79.9)	
		Hispanic White	12 (4)	
		Not Hispanic Not Listed	1 (0.3)	
		Hispanic Not Listed	4 (1.3)	
		Not Hispanic Multiracial	9 (3)	
		Hispanic Multiracial	2 (0.7)	
	**College standing (n=303), n (%)**			
		First-year	53 (17.5)	
		Second-year	55 (18.2)	
		Third-year	105 (34.7)	
		Fourth-year	83 (27.4)	
		Other	7 (2.3)	
	**Residence (n=304), n (%)**			
		Parent’s or relative’s home	17 (5.6)	
		College dorm or residence hall	83 (27.3)	
		House, apartment, or room (not college-affiliated)	202 (66.5)	
		Fraternity or sorority house	2 (0.7)	
	Typical number of drinks per week, mean (SD)^b^			13.14 (7.96)
	Typical number of drinking days per week, mean (SD)^c^			2.77 (1.06)
	Typical number of drinks per drinking day, mean (SD)^d^			4.73 (2.17)
	Binge drinking days per month, mean (SD*)*^e^			5.96 (3.36)
	Alcohol-Induced Blackout Measure, mean (SD)^f^			10.14 (2.89)
	**Blackout drinking frequency (n=304), n (%)**			
		Daily or almost daily	2 (0.7)	
		Weekly	9 (3)	
		Monthly	69 (22.7)	
		At least once in the past year, but less than monthly	224 (73.7)	
	AUDIT^g^, mean (SD)^h^			9.25 (4.04)
	Cognitive ability, mean (SD)^i^			33.03 (6.41)
	Cognitive concerns, mean (SD)^j^			16.78 (6.46)

^a^The age range was 18-25 years.

^b^The range for typical number of drinks per week was 0-76 drinks.

^c^The range for typical number of drinking days per week was 0-7 days.

^d^The range for typical number of drinks per drinking day was 1-19 drinks/day.

^e^The range for binge drinking days per month was 2-18 days.

^f^The range for the Alcohol-Induced Blackout Measure score was 5-20.

^g^AUDIT: Alcohol Use Disorder Identification Test.

^h^The range for the AUDIT score was 2-28.

^i^The range for the cognitive ability score was 16-40.

^j^The range for the cognitive concerns score was 10-47.

## Discussion

### Anticipated Findings

Identifying the acute associations between alcohol use and blackout drinking behavior on next-day cognitive functioning, as well as the day- and person-level characteristics that moderate these associations, will provide critical information about the role of alcohol use on everyday cognitive functioning and highlight subgroups most in need of monitoring and early intervention. Findings will guide prevention and intervention programming in several key ways. Identifying whether and at what levels alcohol use impacts cognitive functioning can be used as an intervention tool: momentary interventions could incorporate timely feedback on young adults’ self-reported prior-day alcohol use and objective, current cognitive functioning. Day-level moderators, such as hangover symptoms, mood, sleep, and prior-day cannabis and other substance use [35,37-40,42] may affect associations, and momentary intervention content can be tailored to participants’ own risk factors and delivered in moments of greatest risk. Similarly, intervention content on typical drinking patterns and personal risk for cognitive concerns may be incorporated in existing brief motivational interventions tailored to young adults. Person-level variables that independently and synergistically impact alcohol use and cognition (eg, sex, alcohol use severity, and general cognitive functioning) [36,41] will identify higher-risk groups in need of targeted interventions.

Specific to examining blackout drinking, there have been several recent calls to include blackout-specific content in preventive interventions [4], as personalized information on typical drinking patterns, including blackout drinking, could maximize intervention impact. For some young adults, blackout drinking is associated with later drinking-related consequences [65]; thus, incorporating objective, deleterious outcomes of blackout drinking may help detect and intervene on problematic alcohol use patterns earlier. Given that brief alcohol interventions are more effective among individuals who have experienced a blackout [66], and interventions incorporating blackouts are estimated to save approximately $500,000 per year in emergency department visits [67], investigating within-person risks of blackouts and other problematic alcohol use behaviors (eg, high-intensity drinking) within this age group appears promising and impactful.

### Limitations

It should be noted that future findings from this exploratory and developmental study should be interpreted with several caveats. First, generalizability to young adults who are not attending college or are attending a 2-year program may be limited as the data were from individuals aged 18 to 25 years currently attending a 4-year institution. Second, participants predominately identified as non-Hispanic White and female sex assigned at birth, which may limit the generalizability of findings to minoritized racial-ethnic groups and to men. Finally, although some of our cognitive assessments are objective performance-based, the majority of our data are based on participants’ self-reports and may be impacted by social desirability or recall bias.

### Conclusions

Overall, the findings will provide critical insight on the impact of alcohol use on daily cognitive functioning and will highlight subgroups and days at highest risk as targets for intervention. Results will position us to expand upon this work to (1) study young adults for a longer duration to determine whether cognitive functioning concerns accumulate over time and predict longer-term alcohol-related and cognitive problems; (2) study mechanisms underlying the link between alcohol use and cognitive functioning, including concurrent structural and functional brain changes; and (3) examine the role of day- (eg, cannabis) and person-level (eg, use severity) on the alcohol–cognitive functioning link.

## Appendix

Multimedia Appendix 1 Peer review report by the Epidemiology, Prevention and Behavior Research Study Section - National Institute on Alcohol Abuse and Alcoholism (AA-2) Review Group - National Institute on Alcohol Abuse and Alcoholism (National Institutes of Health, USA).

## Abbreviations

EMA: ecological momentary assessment
REDCap: Research Electronic Data Capture
