# Supplementary material for: Acute Next-Day Effects of Alcohol Use on Daily Cognitive Functioning Among Young Adults: Protocol for a 21-Day Diary Study
Source: JMIR Res Protoc. 2025 Sep 17;14:e77584. doi: 10.2196/77584 (PMC12489418; doi:10.2196/77584)
Supplement: Multimedia Appendix 1 [file resprot_v14i1e77584_app1.pdf]

SUMMARY STATEMENT
(Privileged Communication)

PROGRAM CONTACT:
Bradley Kerridge
301-827-7493
bradley.kerridge@nih.gov

Release Date: **07/06/2022**
Revised Date:

Application Number: **1 R21 AA030590-01**

Principal Investigators (Listed Alphabetically):

LINDEN-CARMICHAEL, ASHLEY NICOLE (Contact)
MOGLE, JACQUELINE

Applicant Organization: PENNSYLVANIA STATE UNIVERSITY, THE

Review Group: **AA-2**
Epidemiology, Prevention and Behavior Research Study Section

Meeting Date: **06/06/2022**
Council: **OCT 2022**
Requested Start: **09/01/2022**
RFA/PA: **PA20-195**
PCC: **AE K**
Dual PCC: **EB/JAV**
Dual IC(s): **DA**

Project Title: **An Ecological Investigation of Acute Next-Day Effects of Alcohol Use on Daily Cognitive Functioning**
SRG Action: **Impact Score:29**
Next Steps: **Visit https://grants.nih.gov/grants/next_steps.htm**
Human Subjects: **30-Human subjects involved - Certified, no SRG concerns**
Animal Subjects: **10-No live vertebrate animals involved for competing appl.**
Gender: **1A-Both genders, scientifically acceptable**
Minority: **1A-Minorities and non-minorities, scientifically acceptable**
Age: **7A-Only Adults, scientifically acceptable**

Project Year	Direct Costs Requested	Estimated Total Cost
1	150,000	237,284
2	125,000	197,736
TOTAL	275,000	435,020

ADMINISTRATIVE BUDGET NOTE: The budget shown is the requested budget and has not been adjusted to reflect any recommendations made by reviewers. If an award is planned, the costs will be calculated by Institute grants management staff based on the recommendations outlined below in the COMMITTEE BUDGET RECOMMENDATIONS section.

1R21AA030590-01 Linden-Carmichael, Ashley

RESUME AND SUMMARY OF DISCUSSION: The PI of this R21 application proposes to examine next-day effects of any alcohol use on cognitive functioning (episodic memory, executive functioning, working memory) in young adults using ecological momentary assessment (EMA). The focus on elucidating the short-term impact of alcohol consumption at any level via EMA (e.g., any drinking, light drinking, binge drinking, high-intensity drinking, blackout drinking) on cognition is significant and innovative. Dr. Linden-Carmichael has experience in EMA and alcohol assessments which is complemented by Dr. Mogle's expertise in ambulatory cognitive assessments. The study Aims are clearly laid out and are not depended on each other, this increases the study feasibility. The measures included in the study are well described and supported by previous research. The main concern voiced during discussion was a "missed opportunity" in identifying if the next-day effects of a heavy drinking episode persist over time. Further, some reviewers were not clear how the focus on short-term effects of alcohol consumption on cognition differentiates this proposal from previously done work. Other concerns pertained to the "overly optimistic" power analysis especially for testing interaction effects, to lack of consideration for the impact of practice effect, and lack of information on the duration of EMA assessment. Nevertheless, the reviewers believed that this proposal has a potential for a High Impact.

DESCRIPTION (provided by applicant):

Heavy and frequent alcohol use remains a key public health concern, particularly among young adults. Recent experimental evidence has shown that a single heavy drinking event is associated with short-term structural changes in the brain among a sample of young adults and preliminary diary evidence suggests alcohol use may impact concentration and working memory capacity. As young adults are at highest risk of alcohol use relative to any other age group, such immediate structural changes may signal a need for early prevention and intervention efforts, although the long-term impacts of alcohol use on the brain within a sample of young adults remain unknown. A key indicator of longer-term cognitive outcomes is observed functional impacts on young adult behavior in their daily lives. Changes in daily behaviors during this period could negatively impact cognitive health into midlife. Our team aims to collect intensive data across a 21-day period from a sample of 250 young adults to explore the acute, next-day (post-intoxication) effects of alcohol use on cognitive functioning overall, across multiple timeframes, and by type of drinking episode (light drinking, binge drinking, high-intensity drinking, blackout drinking). We also seek to examine day-level and person-level moderators to inform moments and subgroups at greatest risk and in need of early targeted prevention and intervention efforts. Specifically, the current exploratory and developmental R21 has three aims. Aim 1 will (a) examine acute, next-day effects of any alcohol use on cognitive functioning (episodic memory, executive functioning, working memory) and across various time frames (i.e., upon waking only or persisting throughout the day); and (b) examine whether these daily associations differ by type of drinking episode (light drinking, binge drinking, high-intensity drinking, blackout drinking). Aim 2 will examine day-level moderators (hangover symptoms, mood, sleep, prior day cannabis, other substance use) that may buffer or exacerbate day-level associations between alcohol use (any drinking, light drinking, binge drinking, high-intensity drinking, blackout drinking) and cognitive functioning. Finally, Aim 3 will examine person-level variables (sex, baseline alcohol use severity, general cognitive functioning) as moderators of day-level associations between alcohol use (any drinking, light drinking, binge drinking, high-intensity drinking, blackout drinking) and cognitive functioning. By identifying the acute impacts of varying levels of alcohol use intensity on next-day cognitive functioning as well as the day- and person-level characteristics that moderate these associations, our findings will have critical implications for the role

of alcohol use on everyday cognitive functioning and highlight subgroups most in need of monitoring and early intervention. Findings from this exploratory and developmental R21 will also position our team well to ultimately examine whether day-level associations accumulate and are indicative of later alcohol-related and cognition-related problems.

PUBLIC HEALTH RELEVANCE

Heavy and frequent alcohol use remains a key public health concern, particularly among young adults. The proposed work will collect intensive momentary assessment data to examine associations between higher-risk alcohol use episodes (binge, high-intensity, blackout drinking) and multiple indices of next-day (post- intoxication) cognitive functioning. By identifying the acute impacts of varying levels of alcohol use intensity on next-day cognitive functioning as well as the day- and person-level characteristics that moderate these associations, our findings will have critical implications for the role of alcohol use on everyday cognitive functioning and highlight subgroups most in need of monitoring and early intervention.

CRITIQUE 1

Significance:2
Investigator(s):1
Innovation:1
Approach:3
Environment:1

Overall Impact:

The proposed research seeks to investigate the impacts of various levels of alcohol consumption (any; heavy; binge) on next-day cognition among a sample of young adults using ecological momentary assessment. Understanding these short-term impacts is important in understanding the causal chain linking heavy and/or binge alcohol use to longer-term cognitive decline, as well as shorter-term social and functional outcomes in young adults. Investigators propose to recruit young adults in the same city where they are located (a college town in PA) through online advertisements and physical fliers. Interested participants then use a web link to answer screening questions, complete informed consent, and then engage in the 2-week EMA protocol. Measures for alcohol use as well as cognition are strong, with the latter including not only self-assess cognition but also objective cognitive assessments. The PIs both bring substantive expertise in the collection and analysis of EMA studies and have complementary areas of expertise (Linden-Carmichael - intensive longitudinal studies of alcohol use; Mogle – intensive longitudinal studies of cognition.) Proposed analyses overall are appropriate given data structure and research questions. Main concerns center on (1) validity of sample, given no in-person contact with the researchers will take place; and (2) power for analyses, given power analyses as described did not explicitly take into account correlation of observations within subjects. Secondary concerns centered on how past-day and day-of alcohol use measures would be combined and/or harmonized if reports were different, and the stated potential gap of up to a month between initial survey intake and initiation of EMA protocol.

1. Significance:

Strengths

- Well-outlined case for focusing on young adults; prevalence of risky alcohol use behaviors
- Near-term functional cognitive effects of various levels of alcohol use are not well-defined, as outlined by the investigators – important gap to fill
- From what is written, it appears the biggest benefit of the research is in potentially providing a more temporally proximate link between heavy drinking episodes and cognitive impacts, which can then be expanded upon to determine factors or interventions that promote or inhibit recovery from such impacts.

Weaknesses

- Investigators suggest results (especially those of Aim 3) “may ultimately inform targeted and real-time interventions for days and individuals at greatest risk,” but it is unclear to me what such interventions would look like.

2. Investigator(s):

Strengths

- One MPI is a promising, productive New Investigator currently supported by an NIAAA K01
- Experience in conducting EMA studies for both PIs; EMA experience for alcohol use (Linden-Carmichael), and ambulatory cognitive assessments (Mogle)
- Both MPIs have the statistical expertise/experience to implement planned analyses

Weaknesses

- Contribution of Co-I Wilson is unclear

3. Innovation:

Strengths

- The application of the EMA design to cognitive impacts of alcohol use events – and differentiating those events by relative heaviness of drinking – is a strong innovation
- Use of objective assessments of cognition, paired with self-report measures, is also an important innovation
- Tracking cognitive recovery across one (or multiple) day(s) is also an important focus

Weaknesses

- None noted.

4. Approach:

Strengths

- Planned recruitment seems reasonable given both recent past experiences of Linden-Carmichael and the general population focus of the study
- Allowing potential participants to enroll completely virtually will reduce barriers for enrollment and likely increase rapidity of enrollment.

- The proposed EMA strategy is robust. Limiting the daily surveys to ~5minutes will aid with completion and retention. Application of MLE is appropriate given likelihood of missing data across participants.
- Aims are clearly defined, non-dependent on one another, and each make important contributions to the project.
- Analyses overall are clear as described and appropriate given multilevel structure of the data.

Weaknesses

- The onus is on participants contacting the researchers, which will yield a selective sample of drinkers who are willing to participate in such an intensive protocol. Some exploration of the potential biases arising from self-selection are warranted.
- Related, the researchers will not directly interact with the participants in person, making it more possible for fraudulent participation. What steps will the researchers take to (1) verify or increase likelihood of actual age eligibility, and (2) protect against participants enrolling multiple times?
- The investigators state that participants will begin their 21-day intensive participation within a month of starting baseline. Up to a month's gap between completing baseline then participation in the intensive seems like it would increase the chances for participant attrition. Was this same gap present in the prior study by Linden-Carmichael? Why is such a potentially big gap necessary?
- Table one describes substance use measures as including a checklist of all substances used so far that day (for all assessments after first daily report). It is unclear how that will be combined with the first daily report, where participants check off what substances they used the prior day, and for alcohol, number of drinks. What if those do not match? Will the 2nd-4th daily assessments be used to impute alcohol use for missing first daily assessments?
- Power as described by the investigators is adequate, but it is unclear whether power analyses took into consideration non-independence of the observations / within person correlations. I was especially concerned about the potential power of three-way interactions.
- Relatedly, for person-level interactions, given low n's for races other than white, I wonder about the feasibility of conducting such analyses. If planning on combining all "non-white" participants, this may lack validity given variability across such groups in alcohol use behaviors.

5. Environment:

Strengths

- The researchers are affiliated with Penn State University's Edna Bennett Pierce PRC, which provides a well-resourced environment for supporting the proposed research

Weaknesses

- None noted.

Study Timeline:

Strengths

Weaknesses

- None noted.

Protections for Human Subjects

Click Here to Select – Acceptable risks and adequate protections

 Acceptable risks and adequate protections given de-identified data collection procedures.

Data and Safety Monitoring Plan (Applicable for Clinical Trials Only): Click Here to Select – Not applicable

Inclusion Plans

- Sex/Gender: Click Here to Select – Distribution justified scientifically
- Race/Ethnicity: Click Here to Select – Distribution justified scientifically
    - For NIH-Defined Phase III trials, plans for valid design and analysis: Click Here to Select – Not applicable
    - Inclusion/Exclusion Based on Age: Click Here to Select – distribution justified scientifically
    - Comments (Required Unless Not Applicable): Inclusion plan is acceptable given scientific rationale of focus on young adults.

Budget and Period of Support

Recommended budget modifications or possible overlap identified:

- Possible re-examination of or further justification for Dr. Wilson's time on project.

CRITIQUE 2

Significance:3
Investigator(s):1
Innovation:2
Approach:3
Environment:1

Overall Impact:

The present study aims to recruit a sample of 250 young adults with recent histories of heavy drinking and blackout drinking to enroll in a 21-day EMA study where they will be queried regarding memory and cognition. The scientific premise for the study is based on emerging evidence that even single episodes of drinking can impair memory and cognitive function the day after, although it is unclear whether these impairments are associated with any long-term harm. Indeed, the investigators own studies have

demonstrated associations between level of alcohol use with short-term deficits in cognitive functioning. The advance of the present study given the current literature, including the data that the investigators already have available, could be more clarified. It seems an important step to determine whether the next-day effects of a heavy drinking episode persist over time, which could be nicely done in the EMA design but length of heavy drinking impacts on memory and cognition are not proposed in this study. The investigators are highly experienced and well-suited to conduct this work. In terms of the approach, the investigators plan to enroll 250 young adults through convenience sampling and have a track record of high rates of EMA completion. It would be beneficial to the approach to have some understanding of how the investigators plan to include specific subgroups such as under-served racial/ethnic communities, women, or nonbinary and gender nonconforming young adults. The power analysis seemed very optimistic for a sample of 250 people especially for tests of interaction; I assume the investigators powered the study based on assumptions of prevalent exposures and normally distributed outcomes, neither of which may be the case. Finally, the extent to which this study will inform a broader program of research could be more clarified; the investigators generally discuss longer follow-ups or investigations of mechanisms, as well as implications for prevention and intervention, but it is not clear exactly how this study would inform these future studies or prevention measures.

1. Significance:

Strengths

- Heavy drinking and blackouts remain a significant concern for public health, especially among young people
- The effects of heavy drinking on the brain, memory, and cognition are important to study
- An emerging evidence base indicates that even a single episode of heavy drinking can lead to cognitive deficits

Weaknesses

- Without knowing whether memory effects the day after heavy drinking last, or accumulate, etc., it is unclear whether an association observed the day after a heavy drinking episode are important or consequential
- The investigators prior studies have demonstrated associations between level of alcohol use with short-term deficits in cognitive functioning. The advance of the present study given the current literature, including the data that the investigators already have available, could be more clarified.
- It seems an important step to determine whether the next-day effects of a heavy drinking episode persist over time, which could be nicely done in the EMA design but length of heavy drinking impacts on memory and cognition are not proposed in this study.

2. Investigator(s):

Strengths

- The PI is an early-stage investigator with a very strong track record of high impact research, EMA data collection, and in alcohol use and its consequences in young adults
- The remaining team is also strong.

Weaknesses

- None

3. Innovation:

Strengths

- The first study to collect EMA data on next-day memory effects after heavy drinking
- High quality cognitive assessments proposed
- The study plans to separately assess the impact of blackout drinking and heavy drinking

Weaknesses

- Without studying whether next-day effects are temporary, unclear how the present results would inform prevention and intervention

4. Approach:

Strengths

- 250 individuals will be recruited through social media and community ads
- Measures are nicely described, and all reviewed in the literature
- The investigators have a strong track record of high rates of EMA completion
- Preliminary data underscore the team's ability to conduct this work

Weaknesses

- The EMA data collection will be four times per day, but it is not clear whether all measures will be assessed four times a day, or (I assume) only some measures. What is the expected length of time of the EMA surveys?
- It would be beneficial to the approach to have some understanding of how the investigators plan to include specific subgroups such as under-served racial/ethnic communities, women, or nonbinary and gender nonconforming young adults.
- The power analysis seemed very optimistic for a sample of 250 people especially for tests of interaction; I assume the investigators powered the study based on assumptions of prevalent exposures and normally distributed outcomes, neither of which may be the case.

5. Environment:

Strengths

- The environment to conduct the proposed work is supportive and well equipped

Weaknesses

- None

Study Timeline:

Strengths

Weaknesses

- None noted.

Protections for Human Subjects

Acceptable Risks and/or Adequate Protections

Data and Safety Monitoring Plan (Applicable for Clinical Trials Only):

Not Applicable (No Clinical Trials)

Inclusion Plans

- Sex/Gender: Distribution justified scientifically
- Race/Ethnicity: Distribution not justified scientifically
- For NIH-Defined Phase III trials, Plans for valid design and analysis: Not applicable
- Inclusion/Exclusion Based on Age: Distribution justified scientifically
- Investigators state: "We will oversample non-Hispanic/Latinx White participants to ensure adequate representation of underserved populations". Not sure if this is a typo, but it was concerning as the inclusion of non-White participants is already quite low.

Budget and Period of Support

Recommend as Requested

CRITIQUE 3

Significance:3
Investigator(s):2
Innovation:3
Approach:2
Environment:1

Overall Impact:

The overall goal of this new submission is to examine the next-day impact of alcohol use on different facets of cognitive functioning. Using a 21-day EMA design, this study will consider the impact of different levels of alcohol use, day-level moderators such as mood, and per-level variables such as biological sex and general cognitive functioning. Strengths of the application include the expertise of the investigative team and their combined experience in collecting and analyzing EMA data of substance use, the research environment, and the consideration of different facets of cognitive functioning. Weaknesses of the application included some minor issues in the approach.

1. Significance:

Strengths

- Prior evidence that one heavy drinking episode impacts brain structure and potentially cognitive functioning. It is unknown how different levels of prior day drinking impact different facets of cognitive functioning.

Weaknesses

- Unclear how results will advance prevention and intervention programming.

2. Investigator(s):

Strengths

- MPIs Linden-Carmichael and Mogle have necessary experience to carry out data collection and analysis. MPIs have a successful history of collaboration.

Weaknesses

- Dr. Wilson's role in the project is a bit vague. As stated, he is bringing expertise in addiction (which both MPIs have) and combining fMRI and EMA data, but it is unclear how that expertise will be used in the current project.

3. Innovation:

Strengths

- Collect 21-day EMA data to study the impact of different levels of alcohol use on different types of next-day cognitive functioning post

Weaknesses

- None noted.

4. Approach:

Strengths

- Use of appropriate statistical models for EMA data. Have thoughtfully considered how cross-level interactions and issues with missing data will be addressed.
- MPIs have previous history of recruiting heavy alcohol users in this age group for shorter duration EMA studies

Weaknesses

- Unclear if there will be enough individuals reporting black-out episodes for statistical testing. This concern is minor given that this is likely a feasibility study.
- Recruitment and over-sampling strategy and procedures for non-college-attending young adults and racial/ethnic minorities is not described
- Some individuals may still be intoxicated from previous day drinking for some next-day assessments.

5. Environment:

Strengths

- Penn State is an excellent environment to conduct the proposed project. All resources needed are present.

Weaknesses

- None noted.

Study Timeline:

Strengths

Weaknesses

- None noted.

Protections for Human Subjects

Acceptable Risks and/or Adequate Protections

- Minimal risk

Data and Safety Monitoring Plan (Applicable for Clinical Trials Only):

Inclusion Plans

- Sex/Gender: Distribution justified scientifically
- Race/Ethnicity: Distribution justified scientifically
- For NIH-Defined Phase III trials, Plans for valid design and analysis:
- Inclusion/Exclusion Based on Age: Distribution justified scientifically
- Anticipated sex and race/ethnicity breakdowns are anticipated to be similar to local population for this age group.

Resource Sharing Plans

- Acceptable

Budget and Period of Support

Recommend as Requested

THE FOLLOWING SECTIONS WERE PREPARED BY THE SCIENTIFIC REVIEW OFFICER TO SUMMARIZE THE OUTCOME OF DISCUSSIONS OF THE REVIEW COMMITTEE, OR REVIEWERS' WRITTEN CRITIQUES, ON THE FOLLOWING ISSUES:

PROTECTION OF HUMAN SUBJECTS: ACCEPTABLE

INCLUSION OF WOMEN PLAN: ACCEPTABLE

INCLUSION OF MINORITIES PLAN: ACCEPTABLE

INCLUSION ACROSS THE LIFESPAN: ACCEPTABLE

COMMITTEE BUDGET RECOMMENDATIONS: The budget was recommended as requested.

Footnotes for 1 R21 AA030590-01; PI Name: Linden-Carmichael, Ashley Nicole

NIH has modified its policy regarding the receipt of resubmissions (amended applications).See Guide Notice NOT-OD-18-197 at https://grants.nih.gov/grants/guide/notice-files/NOT-OD-18-197.html. The impact/priority score is calculated after discussion of an application by averaging the overall scores (1-9) given by all voting reviewers on the committee and multiplying by 10. The criterion scores are submitted prior to the meeting by the individual reviewers assigned to an application, and are not discussed specifically at the review meeting or calculated into the overall impact score. Some applications also receive a percentile ranking. For details on the review process, see http://grants.nih.gov/grants/peer_review_process.htm#scoring.

MEETING ROSTER

Epidemiology, Prevention and Behavior Research Study Section
National Institute on Alcohol Abuse and Alcoholism Initial Review Group
NATIONAL INSTITUTE ON ALCOHOL ABUSE AND ALCOHOLISM
AA-2
06/06/2022 - 06/07/2022

Notice of NIH Policy to All Applicants: Meeting rosters are provided for information purposes only. Applicant investigators and institutional officials must not communicate directly with study section members about an application before or after the review. Failure to observe this policy will create a serious breach of integrity in the peer review process, and may lead to actions outlined in NOT-OD-22-044 at https://grants.nih.gov/grants/guide/notice-files/NOT-OD-22-044.html, including removal of the application from immediate review.

CHAIRPERSON(S)

MCGUE, MATTHEW K., PHD
PROFESSOR
DEPARTMENT OF PSYCHOLOGY
INSTITUTE OF HUMAN GENETICS
UNIVERSITY OF MINNESOTA
MINNEAPOLIS, MN 55455

MEMBERS

BANDOLI, GRETCHEN E, BS, MPH, PHD *
ASSISTANT PROFESSOR
DEPARTMENT OF PEDIACTRICS
UNIVERSITY OF CALIFORNIA-SAN DIEGO
LA JOLLA, CA 92093

CAETANO, RAUL, PHD, MD
SENIOR RESEARCH SCIENTIST
PREVENTION RESEARCH CENTER
PACIFIC INSTITUTE FOR RESEARCH AND EVALUATION
OAKLAND, CA 94612

CANO, MIGUEL ANGEL, PHD
ASSOCIATE PROFESSOR
DEPARTMENT OF EPIDEMIOLOGY
FLORIDA INTERNATIONAL UNIVERSITY
MIAMI, FL 33199

CARTER, PATRICK MICHAEL, MD *
ASSOCIATE PROFESSOR AND CHAIR
DEPARTMENT OF EMERGENCY MEDICINE
UNIVERSITY OF MICHIGAN
ANN ARBOR, MI 48109

CLARK, SHAUNNA L, PHD *
ASSOCIATE PROFESSOR
DEPARTMENT OF PSYCHIATRY
COLLEGE OF MEDICINE
TEXAS A&M UNIVERSITY
BRYAN, TX 77807

FURR-HOLDEN, C. DEBRA M., PHD
PROFESSOR
DEPARTMENT OF EPIDEMIOLOGY AND BIOSTATISTICS
COLLEGE OF HUMAN MEDICINE
MICHIGAN STATE UNIVERSITY
FLINT, MI 48502

KEYES, KATHERINE MARGARET, PHD
ASSOCIATE PROFESSOR
DEPARTMENT OF EPIDEMIOLOGY
MAILMAN SCHOOL OF PUBLIC HEALTH
COLUMBIA UNIVERSITY
NEW YORK, NY 10032

MADKOUR, AUBREY SPRIGGS, PHD *
ASSOCIATE PROFESSOR
DEPARTMENT OF GLOBAL COMMUNITY HEALTH AND BEHAVIORAL SCIENCE
TULANE UNIVERSITY OF LOUISIANA
NEW ORLEANS, LA 70112

PATRICK, MEGAN ELIZABETH, PHD
RESEARCH PROFESSOR
INSTITUTE FOR SOCIAL RESEARCH
UNIVERSITY OF MICHIGAN
ANN ARBOR, MI 48109

PEDERSEN, ERIC R., PHD *
BEHAVIORAL SCIENTIST
UNIVERSITY OF SOUTHERN CALIFORNIA
LOS ANGELES, CA 90089070

SANCHEZ, MARIANA, PHD
ASSISTANT PROFESSOR
DEPARTMENT OF HEALTH PROMOTION AND DISEASE PREVENTION
FLORIDA INTERNATIONAL UNIVERSITY
MIAMI, FL 33199

SUBICA, ANDREW MAKOTO, PHD
ASSISTANT PROFESSOR
SOCIAL MEDICINE, POPULATION, AND PUBLIC HEALTH
CENTER FOR HEALTHY COMMUNITIES
UNIVERSITY OF CALIFORNIA, RIVERSIDE
RIVERSIDE, CA 92521

TALLEY, AMELIA E, MA, BA, PHD *

TOFIGHI, DAVOOD, PHD *
UNIVERSITY OF NEW MEXICO
DEPARTMENT OF PSYCHOLOGY
ALBUQUERQUE, NM 87131

SCIENTIFIC REVIEW OFFICER

GHAMBARYAN, ANNA, MD, PHD
SCIENTIFIC REVIEW OFFICER
EXTRAMURAL PROJECT REVIEW BRANCH
OFFICE OF EXTRAMURAL ACTIVITIES
NATIONAL INSTITUTE ON ALCOHOL ABUSE AND ALCOHOLISM
NATIONAL INSTITUTES OF HEALTH
BETHESDA, MD 20892

EXTRAMURAL SUPPORT ASSISTANT

STRINGFIELD, DONNA
EXTRAMURAL SUPPORT ASSISTANT
OFFICE OF EXTRAMURAL ACTIVITIES
NATIONAL INSTITUTE ON ALCOHOL ABUSE AND ALCOHOLISM
NATIONAL INSTITUTES OF HEALTH
6700B ROCKLEDGE DRIVE, ROOM 1460B, MSC 6902
BETHESDA, MD 20892

* Temporary Member. For grant applications, temporary members may participate in the entire meeting or may review only selected applications as needed.

Consultants are required to absent themselves from the room during the review of any application if their presence would constitute or appear to constitute a conflict of interest.
